# Enhancing Distress Tolerance Skills in Adolescents With Anorexia Nervosa Through the BALANCE Mobile App: Feasibility and Acceptability Study

**DOI:** 10.2196/70278

**Published:** 2025-02-28

**Authors:** Christina Miranda, Brittany Matheson, Nandini Datta, Aileen Whyte, Hyun-Joon Yang, Paul Schmiedmayer, Vishnu Ravi, Oliver Aalami, James Lock

**Affiliations:** 1 School of Medicine, Stanford University, 291 Campus Dr, Stanford, CA, 94305, United States, 1 9084425567; 2 Department of Psychiatry and Behavioral Sciences, Stanford Medicine, Stanford, CA, United States; 3 Stanford Mussallem Center for Biodesign, Stanford University, Stanford, CA, United States

**Keywords:** mHealth, mobile health, mobile application, emotion regulation, eating disorders, family-based treatment, distress tolerance, mealtimes

## Abstract

**Background:**

Anorexia nervosa is a severe psychiatric disorder with high morbidity and mortality, particularly among adolescents. Family-based treatment (FBT) is the leading evidence-based intervention for adolescent anorexia nervosa, involving parents in renourishment and behavior interruption. Despite its effectiveness, challenges in distress tolerance and emotion regulation during high-stress situations, such as mealtimes, contribute to suboptimal treatment outcomes, with only 35% to 50% of adolescents achieving full recovery. Enhancing distress tolerance skills during FBT may improve treatment responses and recovery rates. The BALANCE mobile app was developed to address this need, offering real-time, dialectical behavior therapy (DBT)–based distress tolerance skills to support adolescents and families during mealtimes.

**Objective:**

Our aim was to explore the feasibility and acceptability of a mobile app designed to deliver distress tolerance skills to adolescents with and adolescents without anorexia nervosa. When fully programmed and optimized, we plan to use the mobile app to improve distress tolerance during mealtimes for adolescents with anorexia nervosa undergoing FBT.

**Methods:**

BALANCE was developed collaboratively with Stanford University’s Center for Biodesign, leveraging the expertise of clinical psychologists and using biodesign student input and the Stanford Spezi ecosystem. The app underwent an iterative development process, with feedback from adolescent users. The initial feasibility and acceptability of the app were assessed through self-reported questionnaires and structured interviews with 24 adolescents aged 12 to 18 years, including 4 diagnosed with anorexia nervosa and 20 healthy controls. Adolescents with anorexia nervosa specifically used the app during mealtimes, and healthy controls used it as needed. Participants assessed the app’s usability, perceived effectiveness, and its impact on their distress tolerance.

**Results:**

The app demonstrated high usability and acceptability. Of 24 participants, 83% (n=20) reported enjoying the app, 88% (n=21) would recommend it to peers, and 100% (n=24) found it user-friendly. Adolescents with anorexia nervosa reported that BALANCE helped them manage stressful mealtimes more effectively, highlighting features such as guided meditation, breathing exercises, and gamification elements as particularly effective. Healthy controls provided additional feedback, confirming the app’s broad appeal to the target audience and potential scalability. Preliminary findings suggest that BALANCE may enhance distress tolerance in adolescents with and adolescents without anorexia nervosa.

**Conclusions:**

BALANCE shows promise as an innovative mobile health intervention for enhancing distress tolerance in adolescents with anorexia nervosa. Its user-friendly design and tailored DBT-based skills make it a feasible tool for integration into FBT. Future research should explore its integration into clinical practice and its impact on treatment outcomes. As distress tolerance skills are relevant to a range of mental health conditions, future research may also expand BALANCE’s application to broader adolescent populations.

## Introduction

Anorexia nervosa is a severe psychiatric disorder that poses significant morbidity and mortality risks, particularly among adolescents. Family-based treatment (FBT) has been shown to be effective in addressing adolescent anorexia nervosa by involving parents to promote renourishment and disrupt eating disorder behaviors [1]. However, despite its success, only 35% to 50% of adolescents fully recover through FBT, highlighting the need for innovative strategies to enhance treatment outcomes [2,3].

Recent research highlights the role of distress tolerance in eating disorder recovery, with studies demonstrating that lower distress tolerance is associated with poorer treatment outcomes [4]. Despite the increasing adoption of mobile health (mHealth) interventions in psychiatric care, few apps specifically target distress tolerance during mealtimes for adolescents with anorexia nervosa. The BALANCE app was developed to address this gap, providing real-time, dialectical behavior therapy (DBT)–based distress tolerance strategies that can be integrated into FBT.

Several studies in the past 2 to 3 years have examined digital interventions for eating disorders, reinforcing the importance of personalized and interactive tools in improving treatment adherence [5-7]. Digital interventions have been found to enhance engagement by providing real-time coping strategies, facilitating adherence to treatment protocols, and supporting long-term behavior change [7]. Additionally, studies emphasize the importance of incorporating structured distress tolerance strategies into existing treatment models to optimize clinical outcomes [4]. By leveraging DBT principles, BALANCE aims to enhance distress tolerance skills, thereby ultimately improving treatment efficacy for adolescents with anorexia nervosa.

This study evaluated the feasibility and acceptability of the BALANCE mobile app, which was designed to improve distress tolerance in adolescents, with the aim of ultimately having the app be used during mealtimes for adolescents with anorexia nervosa undergoing FBT.

## Methods

### Recruitment Process and Procedures

To ensure a systematic and transparent recruitment process, we implemented the following step-by-step procedure: (1) identifying eligible participants, (2) screening for eligibility, (3) obtaining parental and participant consent, and (4) conducting an orientation session.

#### Identification of Eligible Participants

A total of 24 adolescents aged 12 to 18 years were recruited for this study. For the initial iteration of the app, we focused on acceptability and feasibility among the adolescent age group. In total, 20 healthy adolescent controls were recruited from the community through flyers on social media platforms. We also recruited 4 adolescents with anorexia nervosa from the Stanford University Comprehensive Care Program—a 15-bed inpatient unit within Lucile Packard Children’s Hospital that provides multidisciplinary care for medically compromised adolescents with eating disorders—to preliminarily assess acceptability and feasibility with this clinical group.

#### Screening for Eligibility

Interested participants completed an initial phone call with study staff to determine eligibility based on diagnosis, age, and access to a smart device that could download the BALANCE app.

#### Parental and Participant Consent

For participants younger than 18 years, parental consent and adolescent assent were obtained. Participants aged 18 years provided informed consent independently. All participants received detailed study explanations, including explanations about potential risks and benefits.

#### Orientation Session

Eligible participants attended an orientation session where they learned how to use the app, including its features and intended usage during mealtimes.

### Ethical Considerations

This study was approved by Stanford University’s institutional review board, under the protocol titled “Refinement of Emotion Regulation App for Adolescents with Anorexia Nervosa at Mealtimes During Family-Based Treatment” (eProtocol #69550), adhering to ethical guidelines for human subject research. Informed consent was obtained from all participants aged 18 years, and for participants younger than 18 years, consent was secured from their parents or legal guardians alongside adolescent assent. To ensure privacy and confidentiality, all data were deidentified prior to analysis and securely stored on encrypted servers that were accessible only to the research team. Findings were reported in aggregate to prevent individual identification. Participants were not monetarily compensated but were provided early access to the BALANCE app and allowed continued use after this study’s completion.

### App Development and Collaboration

BALANCE was developed in collaboration with Stanford University’s Eating Disorder Research Team and a Stanford biodesign course on mHealth app development [8]. The course enabled computer science undergraduate and graduate students to provide input on app features, interface design, and technical functionality, leveraging the Stanford Spezi open-source digital health framework [9] and student expertise in mHealth technology. Spezi was designed for the rapid development of modern, interoperable mHealth apps based on an ecosystem of modules that encapsulate common functionality and build upon international standards for health data. Prior research on app-based interventions for eating disorders demonstrated the importance of incorporating lived experiences of potential users into the development process [10]. Similarly, the BALANCE app development followed an iterative, user-centered design process, which incorporated 2 waves of feedback from adolescent users (N=10), ensuring the app’s relevance and usability for the target adolescent age group. Licensed clinical psychologists with expertise in DBT-based distress tolerance skills guided the creation of the app’s core content, while students helped refine the user experience through feedback loops and usability testing [11].

**Figure 1.**
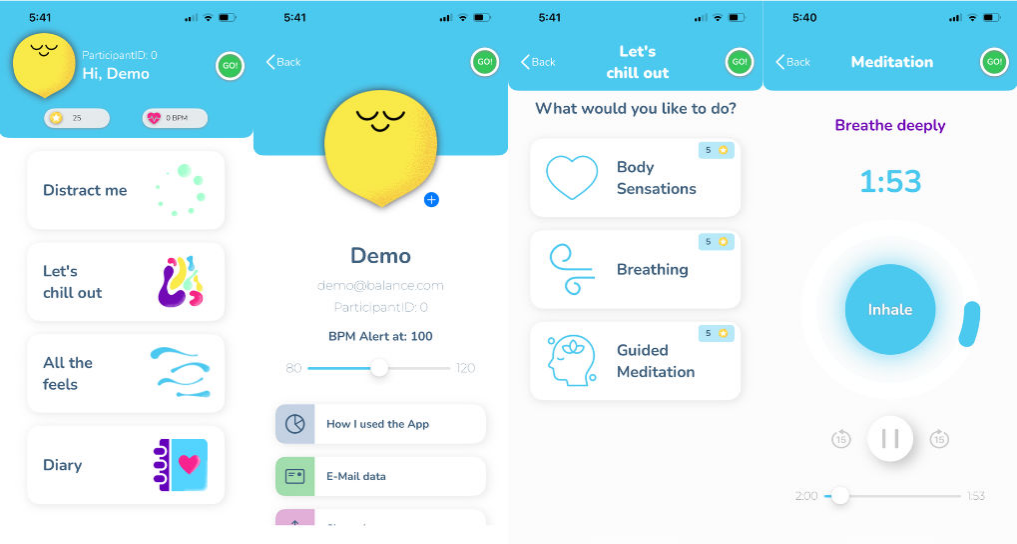
BALANCE app interface and features. This figure showcases the design and core functionalities of the BALANCE app—a mobile health intervention developed to deliver distress tolerance skills to adolescents aged 12 to 18 years. The app incorporates guided meditation, emotion tracking, and gamification features, and it was developed through a collaborative process involving clinical psychologists and biodesign students. Screenshots depict the user interface and interactive elements.

BALANCE incorporates several features designed to improve distress tolerance in adolescents. Users can select a distress tolerance skill, such as distraction, relaxation, or emotion tracking, through the app’s interface (Figure 1). Data on app use (eg, frequency, duration, and skill type) are collected. The app also includes a gamification feature, rewarding users with coins (ie, for customizing an avatar) based on their engagement. The app can be opened directly when prompted by the adolescents or their parents. Additionally, when the app is integrated with a user’s personal smartwatch, notifications are sent to the user when their heart rate increases enough to indicate the possibility of emotional distress, prompting them to open the app. Therapists working with the adolescents in FBT have access to a dashboard that provides information about adolescents’ app use in visual tables and figures. Therapists can integrate this information (eg, how often the app was used, what time of day app use occurred, and what app features were accessed) into FBT when working with the patients and their family.

### Study Design

This study assessed the BALANCE app’s feasibility and acceptability, using self-reported questionnaires and interviews with adolescent users. A secondary aim was to explore app use among adolescents with anorexia nervosa. Healthy adolescents used the app for at least 30 minutes, while adolescents with anorexia nervosa used the app during mealtimes. Measures included usability, perceived effectiveness, and distress tolerance outcomes. Structured interviews allowed participants to elaborate on survey responses to suggest specific modifications to the app, comment on the design, alert us about any usability issues, and share any other feedback that was not captured by survey questions.

### Measures

Participants rated their experience with the app by using Likert-scale surveys and open-ended questions about the app’s functionality, design, and impact on emotion regulation.

## Results

### Participant Demographics

Participants consisted of healthy adolescents (n=20) and adolescents with anorexia nervosa (n=4). Participants were racially and ethnically diverse; of the 24 participants, 16 (67%) identified as female, 8 (33%) identified as male, 13 (54%) identified as White, 8 (33%) identified as Asian, and 3 (13%) identified as Hispanic or Latino.

### Survey Results

The majority of participants found the app user-friendly (n=24, 100%) and helpful (n=22, 92%). Of the 24 participants, 20 (83%) reported enjoying the app, and 21 (88%) would recommend it to a friend. Notably, the guided meditation and breathing exercises were highlighted as particularly helpful during stressful situations. Participant ratings of the app were generally consistent between adolescents with and adolescents without anorexia nervosa (Table 1). Preliminary findings suggest that the BALANCE app improves adolescents’ self-reported ability to manage emotional states. Adolescents with anorexia nervosa reported a decrease in distress and increased ability to tolerate discomfort associated with eating (Tables 1-3).

**Table 1.** Survey responses from the 24 adolescents (4 with anorexia nervosa and 20 healthy controls) on the BALANCE app. Responses (collected from July 2023 to January 2024) highlight user satisfaction, ease of use, and distress tolerance skills.^a^

Survey statement	Strongly disagree, n (%)	Disagree, n (%)	Neutral, n (%)	Agree, n (%)	Strongly agree, n (%)
I enjoyed using this application	0 (0)	0 (0)	0 (0)	20 (83)	4 (17)
This app has functionality that is easy to understand	0 (0)	0 (0)	2 (8)	17 (71)	5 (21)
This app has a user-friendly interface design	0 (0)	0 (0)	0 (0)	14 (58)	10 (42)
This app is exhausting or difficult to use	11 (46)	10 (42)	2 (8)	1 (4)	0 (0)
I would recommend this app to a friend	0 (0)	0 (0)	3 (13)	21 (88)	0 (0)
This app helped me to regulate my emotions	0 (0)	1 (4)	8 (33)	15 (63)	0 (0)
I found this app to be triggering or unhelpful to my emotional state	16 (67)	6 (25)	0 (0)	2 (8)	0 (0)

a Percentages may not add up to 100% due to rounding.

**Table 2.** Survey responses from the 20 healthy adolescent controls on the BALANCE app. Responses (collected from July 2023 to January 2024) highlight user satisfaction, ease of use, and distress tolerance skills.^a^

Survey statement	Strongly disagree, n (%)	Disagree, n (%)	Neutral, n (%)	Agree, n (%)	Strongly agree, n (%)
I enjoyed using this application	0 (0)	0 (0)	0 (0)	16 (80)	4 (20)
This app has functionality that is easy to understand	0 (0)	0 (0)	1 (5)	14 (70)	5 (25)
This app has a user-friendly interface design	0 (0)	0 (0)	0 (0)	12 (60)	8 (40)
This app is exhausting or difficult to use	9 (45)	10 (50)	0 (0)	1 (5)	0 (0)
I would recommend this app to a friend	0 (0)	0 (0)	3 (15)	17 (85)	0 (0)
This app helped me to regulate my emotions	0 (0)	1 (5)	6 (30)	13 (65)	0 (0)
I found this app to be triggering or unhelpful to my emotional state	13 (65)	5 (25)	0 (0)	2 (10)	0 (0)

a Percentages may not add up to 100% due to rounding.

**Table 3.** Survey responses from the 4 adolescents with anorexia nervosa on the BALANCE app. Responses (collected from July 2023 to January 2024) highlight user satisfaction, ease of use, and distress tolerance skills.^a^

Survey statement	Strongly disagree, n (%)	Disagree, n (%)	Neutral, n (%)	Agree, n (%)	Strongly agree, n (%)
I enjoyed using this application	0 (0)	0 (0)	0 (0)	4 (100)	0 (0)
This app has functionality that is easy to understand	0 (0)	0 (0)	1 (25)	3 (75)	0 (0)
This app has a user-friendly interface design	0 (0)	0 (0)	0 (0)	2 (50)	2 (50)
This app is exhausting or difficult to use	2 (50)	0 (0)	2 (50)	0 (0)	0 (0)
I would recommend this app to a friend	0 (0)	0 (0)	0 (0)	4 (100)	0 (0)
This app helped me to regulate my emotions	0 (0)	0 (0)	2 (50)	2 (50)	0 (0)
I found this app to be triggering or unhelpful to my emotional state	3 (75)	1 (25)	0 (0)	0 (0)	0 (0)

a Percentages may not add up to 100% due to rounding.

## Discussion

### Summary of Findings

This study demonstrated the feasibility and acceptability of the BALANCE mobile app for delivering distress tolerance skills to adolescents, including those with anorexia nervosa. The app was well received, with 83% (20/24) of participants reporting enjoyment, 88% (21/24) reporting that they would recommend it to peers, and 100% (24/24) finding it user-friendly. Adolescents with anorexia nervosa reported improvements in their ability to tolerate mealtime distress, suggesting that the app may serve as a valuable adjunct to FBT. These findings align with this study’s primary objective to assess the feasibility and acceptability of the app, paving the way for its integration into clinical practice. Additionally, this study provides initial evidence that BALANCE may help address distress-related barriers to effective meal completion—a crucial factor in anorexia nervosa recovery.

### Interpretations

The BALANCE app provides a structured and scalable solution to a critical gap in FBT by delivering real-time distress tolerance skills for high-stress mealtimes [12,13]. Distress tolerance deficits contribute to poor treatment adherence and increased dropout rates among adolescents undergoing FBT [12,13]. The high usability ratings suggest that BALANCE may effectively supplement traditional therapeutic interventions, thereby improving mealtime coping mechanisms.

Compared to other mHealth apps for eating disorders, BALANCE is unique in its focus on real-time distress tolerance rather than self-monitoring or general emotional regulation [5,7]. Its DBT-based approach aligns with research showing the efficacy of distress tolerance training in improving treatment engagement and emotional resilience in adolescents with anorexia nervosa [14,15]. Gamification and interactive features further support engagement—a crucial factor in digital intervention success [5,6].

As digital tools become more integrated into clinical practice, BALANCE could be incorporated into standard FBT protocols to improve accessibility and engagement. By providing just-in-time intervention during mealtimes, it may help adolescents manage high-anxiety eating situations and support better treatment adherence and long-term recovery.

### Limitations

This study’s limitations include a small sample size, particularly the limited number of adolescents with anorexia nervosa. Additionally, the reliance on self-reported data introduces potential biases, and the short duration of app use prevents long-term effectiveness assessment. Future research should address these limitations by conducting larger-scale trials, exploring longitudinal impacts, and assessing BALANCE’s integration into standard FBT protocols. Investigating potential barriers to app engagement in diverse clinical settings is also necessary for optimizing its implementation.

### Conclusions and Broader Implications

The BALANCE app is a novel, acceptable, and feasible tool for delivering distress tolerance skills to adolescents with anorexia nervosa. It shows promise for use during high-stress mealtimes, addressing a critical barrier to the effectiveness of FBT. The pilot testing of the app’s integration into FBT is underway, assessing its impact on treatment adherence and distress tolerance in real-world clinical settings. If successfully implemented, BALANCE could enhance treatment outcomes, reduce barriers to recovery, and improve FBT effectiveness. Future research should explore its broader applicability to other mental health conditions wherein distress tolerance plays a key role, to ensure its scalability and long-term benefits for adolescent mental health interventions.

## Abbreviations

DBT: dialectical behavior therapy
FBT: family-based treatment
mHealth: mobile health
